# The genetic diversity and geographical separation study of *Oncomelania hupensis* populations in mainland China using microsatellite loci

**DOI:** 10.1186/s13071-016-1321-z

**Published:** 2016-01-20

**Authors:** Wei Guan, Shi-Zhu Li, Eniola Michael Abe, Bonnie L. Webster, David Rollinson, Xiao-Nong Zhou

**Affiliations:** National Institute of Parasitic Diseases, Chinese Center for Disease Control and Prevention, Shanghai, 200025 People’s Republic of China; Key Laboratory of Parasite and Vector Biology, Ministry of Health, WHO Collaborating Center for Malaria, Schistosomiasis and Filariasis, Shanghai, 200025 People’s Republic of China; Department of Zoology, Federal University Lafia, P.M. B 146, Lafia, Nasarawa State Nigeria; Wolfson Wellcome Biomedical Laboratories, Department of Zoology, Natural History Museum, Cromwell Road, London, SW7 5BD UK

**Keywords:** *Oncomelania hupensis*, *Schistosoma japonicum*, Microsatellites DNA, Polymorphism, Genetic differentiation

## Abstract

**Background:**

*Oncomelania hupensis* is the unique intermediate host of *Schistosoma japonicum*, which plays a crucial role in the transmission of schistosomiasis. The endemic area of *S. japonicum* is strictly consistent with the geographical distribution of *O. hupensis*.

**Methods:**

A total of 24 populations of *O. hupensis* from four ecological landscapes were selected for analysis of genetic diversity by screening eight microsatellite DNA polymorphic loci.

**Results:**

The number of alleles per locus ranged from 29 to 70 with an average of 45.625 and that of effective alleles were 18.5 to 45.8 with an average of 27.4. The observed (*Ho*) and expected (*He*) heterozygosities varied from 0.331 to 0.57 and from 0.888 to 0.974, respectively. The mean of polymorphism information content *(PIC)* for all populations was 0.940, appearing polymorphic for all loci. For the fixation index of F-Statistics, *Fit* and *Fst* were 54.95 and 37.62 %, respectively. Variation of *O. hupensis* chiefly exists among individuals, accounting for 60.58 % of the total variation determined by Analysis of Molecular Variation (AMOVA). Variation among individuals within populations, among populations within groups and among groups only accounted for 26.60, 8.04 and 4.78 %, respectively. This distribution of variation suggests that genetic differences principally originate from within-populations rather than among-populations. Moreover, UPGMA cluster analysis showed that the populations spreading within middle and lower reaches of the Yangtze River (HBWH, JSYZ, JXNC, HNHS, JXJJ, AHWW, HBJL, JXDC, HNNX, JSYZJZ, ZJJH, AHNG and AHWJ) clustered together first, then gathered with the populations in the high mountains (SCMS, SCYA, SCPJ, YNEY, SCLS, YNWS and SCXC), coastal hills (FJFQ and FJFZ) and Karst landform (GXBS and GXYZ) successively.

**Conclusion:**

This study provides novel insight into the theoretical source of genetic differentiation of *Oncomelania hupensis* in mainland China, which is critical for the epidemiological investigation and surveillance of *S. japonicum*.

## Background

Schistosomiasis, caused by *Schistosoma japonicum*, remains one of the most prevalent parasitic diseases and effects severe socio-economic and public health losses in China [1, 2]. *Oncomelania hupensis* is the unique intermediate host of *S. japonicum*, which plays a critical role in the transmission of Schistosomiasis japonica [1, 3]. The geographical distribution of *O. hupensis* coincides with the endemic area of *S. japonicum* [4], which is mainly found throughout the southern region of the Yangtze River basin [5, 6]. As a result, significant genetic differentiation leads to the formation of multiple geographical populations of *O. hupensis* [3]. Coincident with the endemic area for schistosomiasis, *O. hupensis* has been mainly found in four types of ecological landscapes giving rise to subspecies including:(1) *O. h. hupensis* largely in the middle and lower reaches of the Yangtze River (among the provinces of Hunan, Hubei, Jiangxi, Anhui, Jiangsu and Zhejiang) (2) *O. h. robertsoni* in the mountainous region of Sichuan and Yunnan provinces (3) *O. h. guangxiensis* in the Karst landscape of Guangxi province and (4) *O. h. tangi* in the southeastern coastal region of Fujian province [7, 8]. Interestingly, obvious morphological differences have been identified among individuals from the same regional population [9–11]. For example, *O. hupensis* from upstream of Miaohe basin, which contains regions of swamps and lakes, have a ribbed shell while those from downstream have a smooth shell [12].

Microsatellite DNA, known as short tandem repeat (STR) or simple sequence repeat(SSR), occurs throughout the eukaryotic genome. Differences in repetitive sequence numbers allow for high polymorphism due to the ubiquitous occurrence, high copy numbers, high heterozygosity and easy detection within population [13]. Along with other genome mark technology, it has been widely applied to research examining genetic diversity and serves as an important molecular marker [14–17]. At present, microsatellites have been isolated from many different organisms [18–20]. Specifically, from 128 molluscs, a total of 3, 284 microsatellite sequences have been identified [21]. Although the microsatellite DNA library of *O. hupensis* was built recently [22], the microsatellite markers have not been used extensively in population genetic structure studies and genome mapping of *O. hupensis* in P.R. China [23–25]. To deepen our knowledge on the genetic diversity of the intermediate host snail, we developed a novel multiplex PCR method to screen and analyze the genetic diversity of *O. hupensis* using microsatellites loci among the four various ecological landscape populations in mainland China.

## Methods

### Snail sampling

**Fig. 1:**
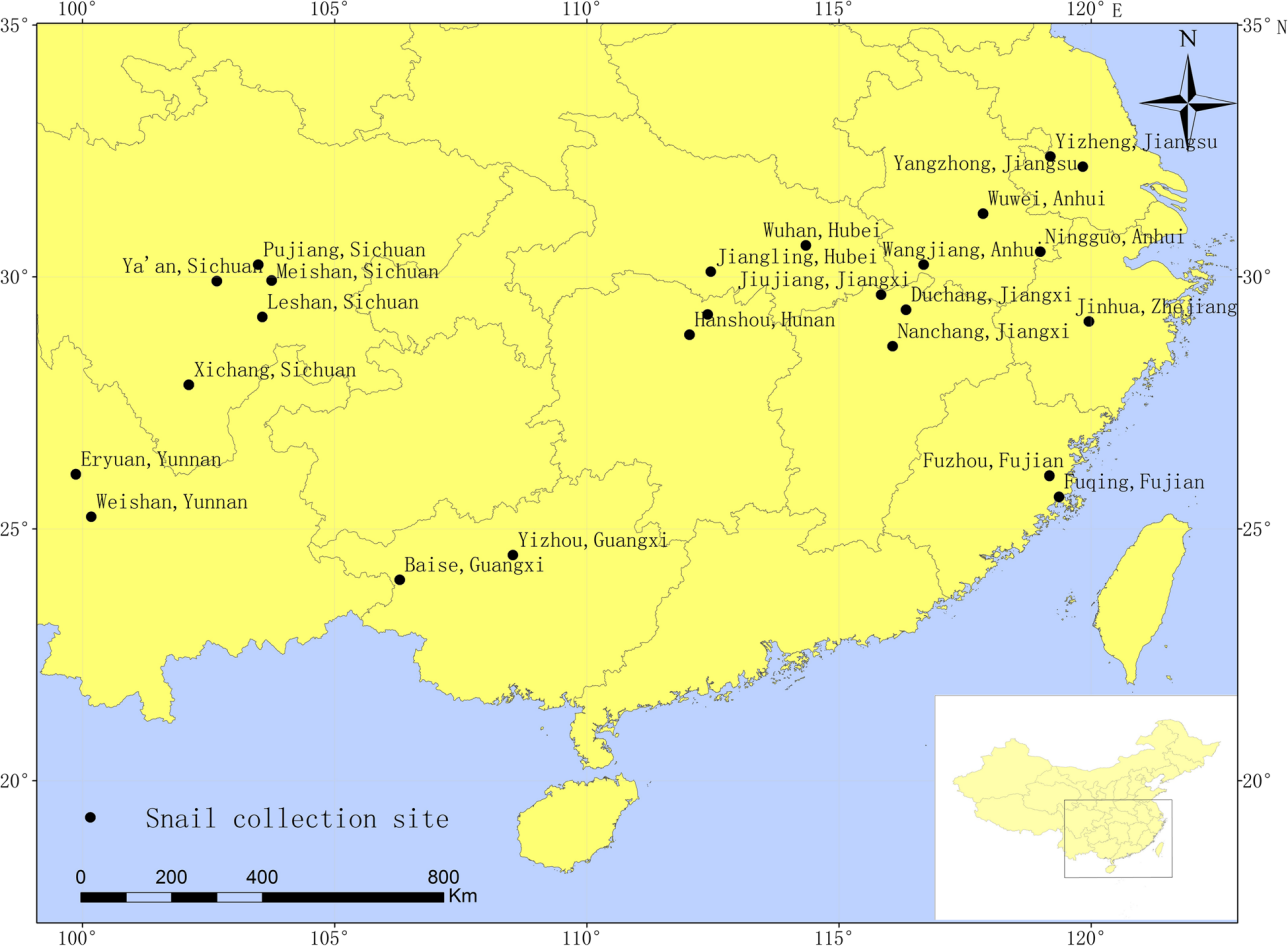
Illustration of geographical location of *O. hupensis* collection sites

A total of 24 populations of *O. hupensis* were sampled from four ecological landscape populations in mainland China covering: (1) the region of swamps and lakes in the middle and lower reaches of the Yangtze River, (2) the mountainous region of the Sichuan and Yunnan provinces, (3) the littoral hill part of the Fujian province and (4) the karst landscape of Guangxi autonomous region (Fig. 1, Table 1).

**Table 1:** Location of *O. hupensis* collection

Collection site(Code)	Geomorphic feature	No. samples	Collection date	Longitude	Latitude
Ningguo, Anhui(AHNG)	swamps and lakes	17	09/12/2012	30.5022° N	118.9891° E
Wangjiang, Anhuui(AHWJ)	swamps and lakes	20	09/12/2012	30.2423° N	116.2814° E
Wuwei, Anhui(AHWW)	swamps and lakes	18	09/12/2012	31.2571° N	117.8573° E
Jiangling, Hubei(HBJL)	swamps and lakes	18	06/14/2013	31.1034° N	112.4631° E
Wuhan, Hubei(HBWH)	swamps and lakes	17	05/11/2012	30.6749° N	114.3865° E
Hanshou, Hunan(HNHS)	swamps and lakes	16	03/18/2013	28.8592° N	112.0378° E
Nanxian, Hunan(HNNX)	swamps and lakes	11	03/18/2013	29.2581° N	112.3972° E
Yizheng,Jiangsu(JSYZ)	swamps and lakes	19	04/21/2013	32.3911° N	119.1914° E
Yangzhong, Jiangsu(JSYZ)	swamps and lakes	18	04/21/2013	32.1942° N	119.8353° E
Duchang, Jiangxi(JXDC)	swamps and lakes	19	04/14/2012	29.3562° N	116.3324° E
Jiujiang, Jiangxi(JXJJ)	swamps and lakes	15	04/14/2012	29.6517° N	115.8356 °E
Nanchang, Jiangxi(JXNC)	swamps and lakes	14	04/14/2012	28.6252° N	116.0642°E
Jinhua, Zhejiang(ZJJH)	swamps and lakes	16	06/23/2012	29.1044° N	120.0052° E
Yaan, Sichuan(SCYA)	Mountains	17	09/25/2012	29.8931° N	102.6651° E
Leshan, Sichuan(SCLS)	Mountains	16	09/25/2012	29.1722° N	103.5759° E
Meishan, Sichuan(SCMS)	Mountains	19	09/25/2012	29.8788° N	104.0949° E
Xichang, Sichuan(SCXC)	Mountains	20	09/27/2012	27.8632° N	102.1134° E
Pujiang, Sichuan(SCPJ)	Mountains	15	09/27/2012	30.2412° N	103.4897° E
Eryuan, Yunnan(YNEY)	Mountains	15	03/21/2013	26.0852° N	112.0371° E
Weishan, Yunnan(YNWS)	Mountains	12	03/21/2013	31.2573° N	117.8574° E
Baise, Guangxi(GXBS)	Karst	9	03/22/2013	23.9829° N	106.1678° E
Yizhou, Guangxi(GXYZ)	Karst	18	03/22/2013	24.4792° N	108.5362° E
Fuqing, Fujian/ FJFQ)	Coastal hills	20	04/17/2012	25.6374° N	119.3652° E
Fuzhou, Fujian(FJFZ)	Coastal hills	17	04/17/2012	25.9911° N	119.1674° E

### DNA preparation

Ten to 20 *O. hupensis* samples were randomly chosen from each site, fed for 1 week and identified as infected or non-infected with *S. japonicum* by observation of cercariae emerging from the snails. Only non-infected snails were used in this study. After removal of the gut and digestive glands from the soft parts of the snails, the 30 mg muscle tissues from the pleopod of a single snail were digested for 3 hours at 56 °C with proteinase K (Amresco Inc. Solon, OH, USA) followed by the standard DNA extraction procedure [26] using mollusc DNA Kit (Omega, USA).

### PCR amplification and detection of PCR products

The microsatellite DNA polymorphic loci were selected and evaluated from previous microsatellite loci library [22]. Two rounds of multiplex PCR reaction were developed including four microsatellite loci in each one, which were identified by different lengths and fluorescence peaks of 6-FAM, VIC, NED and PET labeled by (Sigma-aldrich London, UK). Primer sequences and information are summarized in Table 2.

**Table 2:** Primers of the 8 microsatellite loci in *O. hupensis*

Locus	Primer sequence (5′ → 3′)	Repeat motif	Annealing tempreture/(°C)	Allele size from field snails (bp)	NO. of mutilplex PCR	GenBank accession No.
T1-10	Pf: TCACTCGGGTGTAATGCT	(GA)_38_	55	173–259	1	GU204080
	Pr: TTTGTTACTGATGGTGGC					
T4-25	Pf: CAATAGTTCGACTCGGAAGA	(CT)_35_	52	142–228	1	GU204084
	Pr: CGAGGTATGGCGTTGCTT					
T4-22	Pf: TATCCAAGAAGCCGAAAC	(CA)_10_	50	224–256	1	GU204083
	Pr: GAGGAAAGCGAGGTAAGA					
D11	Pf: TTCAGTTGTCTTATTTCGTG	(TG)_17_	55	141–192	1	GU204223
	Pr: TAGATGTTCACTGGTTTGTC					
T5-11	Pf: ACGCCAGTCTTGGTGTCA	(GT)_14_	55	153–210	2	GU204092
	Pr: TACTTGGGCAGAAGGGTT					
T6-17	Pf: GCTGTCCTTTTACCAACTGC	(AC)_8_	55	192–248	2	GU204108
	Pr: TATCAAAGGATTATGCCGAG					
A18	Pf: GCCGATGATACAAGACCC	(CT)_18_	60	131–256	2	GU204047
	Pr: GAGAATCTCCAGGCACGC					
C22	Pf: CGGTACATCTGGATAGTGG	(CA)_21_	62	185–239	2	GU204145
	Pr: TGCGAAACAGTTGCAGACAC					

The multiplex PCRs were developed using the Type-it Microsatellite PCR Kit (QiaGen, London, UK) with a 25 μl reaction system, including 2x Type-it Multiplex PCR Master Mix 12.5 μl, 10x primer mix 2 μl including four primers in each mix, template DNA 2 μl with less than 200 ng then add RNase-free water to 25 μl. The reaction conditions for PCR amplification were as follows: 95 °C, 5 min; 95 °C, 30 s, 60 °C, 60 s; 72 °C, 30 s, 30 cycles; 65 °C, 30 min for final extension. 1 μl of the PCR product was mixed with 0.6 μl of ROX and 8.4 μl ultrapure Hi-Di formamide, denatured at 95 °C for 5 min and detected using automatic genetic analyzer (3730XL, ABI, USA).

### Analysis of microsatellite diversity

The accurate length of amplified fragments of microsatellite DNA loci were determined using Geneious software(Version 7.0.6) and subsequently exported as an Excel table. The raw data in the table were converted into a recognized format by Arlequin and Genepop using the toolkit of the Excel microsatellite toolkit. The data format which fits for Popgene were acquired by DataTrans 1.0. Various parameters of genetic difference within populations include: number of alleles (Na), number of efficient alleles (Ne), inbreeding coefficient (Fis), expected heterozygosity (He) and observed heterozygosity (Ho) were calculated. The degree of Hardy-Weinberg equilibrium (HWE) and linkage disequilibrium (LD) were tested with Genepop 4.1.10. The frequency of null alleles within every population was calculated in Genepop. The index of genetic variation between populations (Fst), gene flow (Nm) and genetic distance [Fst/ (1-Fst)] were determined using Arlequin [27]. The correlation between genetic distance and geographical distance were tested with Mantel regression. Analysis of molecular variance (AMOVA) was processed through Popgene software, clustering analysis was determined by unweighted pair group method with arithmetic means (UPGMA) and the phylogenetic tree was modified with TreeView [28]. The polymorphism information content (PIC) was calculated according to the formula previously described [28].

## Results

### Gene scan

From the 24 populations of *O. hupensis* sampled, 396 specimens were scanned at the genetic level across eight polymorphic loci of microsatellite DNA. The lengths of amplified fragments for a total of 6,196 microsatellite DNA loci were obtained.

### Genetic differences within populations

Results obtained from the analysis of the 24 populations of *O. hupensis* showed that the number of alleles per locus ranged from 29 to 70 with an average of 45.625, and that of effective alleles were 18.5 to 45.8 with an average of 27.4. The GXYZ and HNHS populations had the minimum and maximum average *Na* values, respectively. The average *He* within populations ranged from 0.888 to 0.974, and the average *Ho* ranged from 0.331 to 0.57. The populations with the highest and lowest *Ho* values were HNHS and GXYZ, respectively. The average *PIC* for all populations of *O. hupensis* was 0.940 (Tables 3, 4 and 5).

**Table 3:** Coefficients of genetic diversity of *O. hupensis* at different loci (the populations of landscape of swamps and lakes)

Populations	Index	Microsatellite loci								Total
		T1-10	T4-25	D11	T4-22	T5-11	T6-27	A18	C22	
AHNG	*Na*	13	12	7	8	14	9	11	10	10.500
	*He*	0.863	0.815	0.806	0.774	0.927*	0.847*	0.929*	0.941*	0.863
	*Ho*	0.412	0.706	0.188	0.706	0.882	0.588	0.071	0.222	0.472
	*PIC*	0.948	0.938	0.913	0.902	0.927	0.932	0.948	0.949	0.932
AHWJ	*Na*	13	15	4	2	8	6	8	1	7.125
	*He*	0.918*	0.936	0.406	0.258	0.749	0.549	0.777	0.000	0.574
	*Ho*	0.588	0.471	0.000	0.059	0.765	0.133	0.200	0.104	0.317
	*PIC*	0.967	0.927	0.987	0.923	0.937	0.927	0.914	0.972	0.944
AHWW	*Na*	6	21	9	10	11	10	15	17	12.375
	*He*	0.810	0.963*	0.856	0.860	0.898	0.849	0.914*	0.936	0.886
	*Ho*	0.091	0.444	0.353	0.278	0.389	0.611	0.412	0.647	0.403
	*PIC*	0.943	0.923	0.938	0.912	0.924	0.972	0.916	0.976	0.937
HBJL	*Na*	12	19	15	10	12	14	13	13	13.500
	*He*	0.913*	0.961*	0.939	0.904	0.879	0.938*	0.895	0.930	0.920
	*Ho*	0.417	0.647	0.357	0.294	0.471	0.706	0.750	0.529	0.521
	*PIC*	0.947	0.933	0.937	0.890	0.927	0.928	0.968	0.972	0.939
HBWH	*Na*	12	19	16	12	15	13	19	18	15.500
	*He*	0.944	0.961*	0.956*	0.903	0.949*	0.924	0.966*	0.966*	0.946
	*Ho*	0.272	0.533	0.467	0.667	0.533	0.733	0.733	0.600	0.567
	*PIC*	0.991	0.896	0.922	0.917	0.958	0.921	0.970	0.927	0.938
HNHS	*Na*	16	21	15	16	17	8	20	18	16.375
	*He*	0.952*	0.974*	0.927	0.907*	0.952*	0.798	0.962*	0.956*	0.929
	*Ho*	0.250	0.750	0.438	0.813	0.733	0.750	0.500	0.688	0.615
	*PIC*	0.956	0.973	0.974	0.932	0.941	0.931	0.952	0.938	0.950
HNNX	*Na*	7	10	7	6	9	9	12	10	8.750
	*He*	0.801	0.913	0.853	0.844*	0.887	0.810	0.942*	0.892	0.868
	*Ho*	0.091	0.818	0.200	0.364	0.636	0.545	0.500	0.909	0.508
	*PIC*	0.936	0.976	0.926	0.927	0.956	0.912	0.951	0.936	0.941
JSYZ	*Na*	7	18	10	12	13	10	12	13	11.875
	*He*	0.909*	0.961*	0.806	0.924*	0.926	0.905*	0.915	0.937	0.910
	*Ho*	0.333	0.733	0.385	0.667	0.500	0.500	0.143	0.571	0.479
	*PIC*	0.897	0.918	0.973	0.899	0.973	0.948	0.940	0.918	0.933
JSYZJZ	*Na*	6	21	8	13	16	11	18	17	13.750
	*He*	0.817	0.954*	0.859	0.894	0.910	0.889*	0.943*	0.938	0.901
	*Ho*	0.111	0.722	0.412	0.611	0.500	0.611	0.500	0.611	0.510
	*PIC*	0.949	0.972	0.936	0.879	0.910	0.980	0.938	0.938	0.938
JXDC	*Na*	7	21	7	11	16	10	12	14	12.250
	*He*	0.890	0.968*	0.800	0.890	0.945*	0.761	0.908	0.922	0.886
	*Ho*	0.143	0.733	0.385	0.467	0.867	0.533	0.133	0.667	0.491
	*PIC*	0.982	0.936	0.926	0.919	0.928	0.979	0.914	0.935	0.943
JXJJ	*Na*	5	14	8	9	11	7	11	16	10.125
	*He*	0.803	0.957*	0.902	0.887	0.931	0.481	0.950*	0.957*	0.859
	*Ho*	0.167	0.545	0.667	0.636	0.727	0.455	0.500	0.818	0.564
	*PIC*	0.968	0.973	0.927	0.898	0.918	0.977	0.927	0.963	0.947
JXNC	*Na*	6	17	9	8	9	7	7	12	9.375
	*He*	0.911*	0.993*	0.908*	0.869*	0.915	0.824	0.856	0.948	0.903
	*Ho*	0.200	0.889	0.500	0.333	0.444	0.778	0.111	0.667	0.490
	*PIC*	0.953	0.911	0.890	0.915	0.937	0.967	0.917	0.967	0.932
ZJJH	*Na*	3	14	1	7	16	0	12	6	7.375
	*He*	0.800	0.940*	0.000	0.764	0.948*	0.000	0.915	0.720	0.636
	*Ho*	0.000	0.625	-	0.563	0.813	-	0.500	0.438	0.490
	*PIC*	0.946	0.927	0.917	0.908	0.918	0.952	0.978	0.962	0.939

- Relevant data unavailable

*Statistically significant deviation from Hardy-Weinberg equilibrium (*P* < 0.01)

**Table 4:** Coefficients of genetic diversity of *O. hupensis* at different loci (the populations of landscape of mountains)

Populations	Index	Microsatellite loci								Total
		T1-10	T4-25	D11	T4-22	T5-11	T6-27	A18	C22	
SCLS	*Na*	13	12	7	8	14	9	11	10	10.500
	*He*	0.863	0.815	0.806	0.774	0.927	0.847	0.929	0.941*	0.863
	*Ho*	0.412	0.706	0.188	0.706	0.882	0.588	0.071	0.222	0.472
	*PIC*	0.948	0.927	0.971	0.909	0.929	0.972	0.927	0.938	0.945
SCMS	*Na*	15	15	12	10	16	10	21	14	14.125
	*He*	0.925*	0.924	0.892	0.863	0.941	0.865	0.964*	0.899	0.909
	*Ho*	0.563	0.700	0.474	0.263	0.850	0.350	0.550	0.650	0.550
	*PIC*	0.983	0.924	0.912	0.965	0.901	0.908	0.967	0.961	0.944
SCPJ	*Na*	6	9	6	3	8	5	9	2	6.000
	*He*	0.748	0.883	0.800	0.446	0.763	0.580	0.742	0.667	0.704
	*Ho*	0.308	0.769	0.385	0.077	0.538	0.500	0.385	0.000	0.370
	*PIC*	0.981	0.959	0.923	0.932	0.972	0.971	0.927	0.940	0.951
SCXC	*Na*	3	8	4	2	4	1	4	5	3.875
	*He*	0.567	0.816	0.743	0.067	0.395	0.000	0.559	0.618	0.471
	*Ho*	0.000	0.467	0.800	0.067	0.400	-	0.067	0.733	0.362
	*PIC*	0.974	0.979	0.890	0.910	0.969	0.918	0.976	0.978	0.949
SCYA	*Na*	9	13	5	3	6	4	7	0	5.875
	*He*	0.869*	0.909	0.756	0.536	0.732	0.538	0.802	0.000	0.643
	*Ho*	0.688	0.938	0.750	0.267	0.375	0.500	0.250	-	0.538
	*PIC*	0.916	0.928	0.910	0.912	0.890	0.935	0.979	0.966	0.957
YNEY	*Na*	8	9	0	4	3	2	4	1	3.875
	*He*	0.818	0.846	0.000	0.251	0.191	0.667	0.251	0.000	0.378
	*Ho*	0.133	0.333	-	0.133	0.067	0.000	0.067	-	0.107
	*PIC*	0.972	0.899	0.926	0.930	0.929	0.927	0.972	0.967	0.941
YNWS	*Na*	6	8	6	2	7	6	7	1	5.375
	*He*	0.779	0.862	0.801	0.159	0.833	0.500	0.848	0.000	0.598
	*Ho*	0.333	0.750	0.500	0.000	0.667	0.417	0.727	-	0.485
	*PIC*	0.954	0.901	0.927	0.915	0.928	0.926	0.981	0.958	0.946

- Relevant data unavailable

*Statistically significant deviation from Hardy-Weinberg equilibrium (*P* < 0.01)

**Table 5:** Coefficients of genetic diversity of *O. hupensis* at different loci (the populations of landscape of karst and coastal hills)

Populations	Index	Microsatellite loci								Total
		T1-10	T4-25	D11	T4-22	T5-11	T6-27	A18	C22	
GXBS	*Na*	0	3	4	2	4	3	3	5	3.000
	*He*	0.000	0.601	0.739	0.667	0.788	0.503	0.582	0.739*	0.577
	*Ho*	-	0.556	0.444	0.000	0.000	0.667	0.111	0.222	0.286
	*PIC*	0.957	0.898	0.918	0.904	0.944	0.920	0.972	0.971	0.936
GXYZ	*Na*	3	1	1	0	0	1	2	1	1.25
	*He*	0.506	0.000	0.000	0.000	0.000	0.000	0.315	0.000	0.103
	*Ho*	0.063	-	-	-	-	-	0.375	-	0.055
	*PIC*	0.946	0.912	0.937	0.901	0.891	0.921	0.969	0.964	0.931
FJFZ	*Na*	9	8	7	1	5	5	6	0	5.125
	*He*	0.861	0.698	0.861	0.000	0.705	0.714	0.754	0.000	0.574
	*Ho*	0.364	0.692	0.364	-	0.538	0.769	0.231	-	0.493
	*PIC*	0.947	0.914	0.925	0.921	0.923	0.931	0.902	0.978	0.930
FJFQ	*Na*	10	10	6	5	12	4	13	6	8.250
	*He*	0.786	0.832	0.864	0.498	0.826	0.800*	0.805	0.377	0.724
	*Ho*	0.222	0.158	0.167	0.444	0.842	0.667	0.842	0.053	0.424
	*PIC*	0.886	0.960	0.927	0.908	0.922	0.907	0.908	0.922	0.918

- Relevant data unavailable

*Statistically significant deviation from Hardy-Weinberg equilibrium (*P* < 0.01)

Significant deviation from Hardy-Weinberg equilibrium (HWE) was observed: 47 out of 192 (24.48 %) possible single exact locus tests (*P* < 0.01).No significant linkage disequilibrium was found between all pairs of the eight loci examined (*P* < 0.01), which indicated the independent behaviour of all loci. Analysis with Genepop software showed the possible occurrence of null alleles, which may lead to deviations from HWE and result in exaggerated levels of genetic differentiation [26, 29, 30]. Null alleles may be due to flank sequence variation decreasing primer annealing efficiency, allele drop out or DNA quality [23, 31].

### Genetic differences among individuals

*Fit* and *Fst* values were 54.95 and 37.62 %, respectively. This suggests that genetic differences mainly exist within populations rather than among those with unbalanced differentiation degrees (Table 6).

**Table 6:** F-Statistics and gene flow for all loci

Locus	Sample Size	*Fis*	*Fit*	*Fst*	*Nm*
T1-10	396	0.6107	0.7534	0.3665	0.4321
T4-25	396	0.0569	0.3253	0.2846	0.6284
D11	396	0.3852	0.6297	0.3977	0.3786
T4-22	396	0.3883	0.6821	0.4803	0.2705
T5-11	396	0.0883	0.3750	0.3144	0.5451
T6-27	396	−0.0044	0.4410	0.4435	0.3138
A18	396	0.4368	0.6229	0.3304	0.5067
C22	396	0.2437	0.5459	0.3996	0.3756
Mean	396	0.2721	0.5459	0.3762	0.4146

**Fig. 2:**
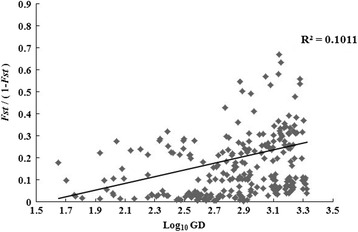
Analysis on the relationship between genetic distance and geographic distance

Mantel^’^s test of regression showed that the correlation (41.97 %) between geographic distance and genetic distance among populations is positive (R^2^ = 0.1011, *P* < 0.05) and genetic distribution of all populations accorded with the Isolation-by-distance Model (Fig. 2, Tables 7 and 8).

**Table 7:**
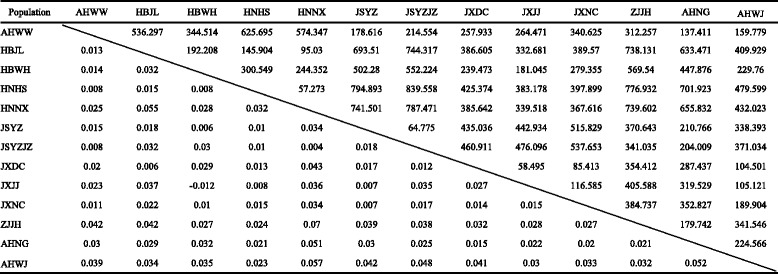
*FST* and geographic distance among paired *O. hupensis* populations of landscape of swamps and lakes

Lower triangule and upper triangule represent *Fst* and geographic distance (GD) / km, respectively

**Table 8:**
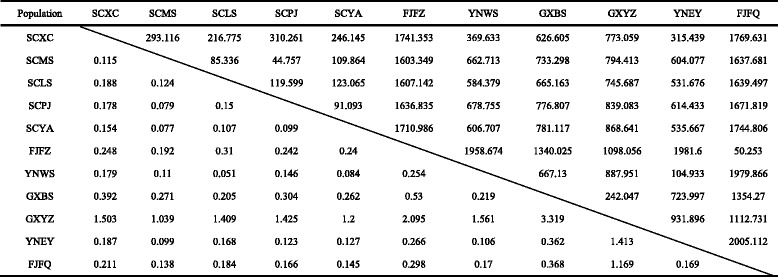
*FST* and geographic distance among paired *O. hupensis* populations of landscape of mountains, karst and Coastal hills

Lower triangule and upper triangule represent *Fst* and geographic distance (GD) / km, respectively

Genetic parameters of the four groups from different landscapes (i.e. lakes and marshes, high mountains, Karst and coastal Hills) showed that *Na* ranged from 2.063 to 11.452, *He* from 0.465 to 0.852 and *Ho* from 0.274 to 0.492. The group from the Karst landscape had the lowest value in all three indices, which indicated its low differentiation degree. AMOVA displayed that variations of *O. hupensis* mainly exists among individuals, which accounted for 60.58 % of total variations, and that of among individuals within populations, among populations within groups and among groups were only 26.60, 8.04 and 4.78 %, respectively (Table 9). This suggests that there is no significant genetic differentiation among groups.

**Table 9:** Analysis of molecular variance (AMOVA) for the *Oncomelania hupensis*

Source of variation	Degree of freedom	Sum of squares	Variance components	Percentage of variation/%
Among group	3	15.653	0.02386	4.78
Among populations within groups	20	35.115	0.04015	8.04
Among individuals within populations	333	189.196	0.13282	26.60
Within individuals	357	108.000	0.30252	60.58
Total	713	347.964	0.49935	

**Fig. 3:**
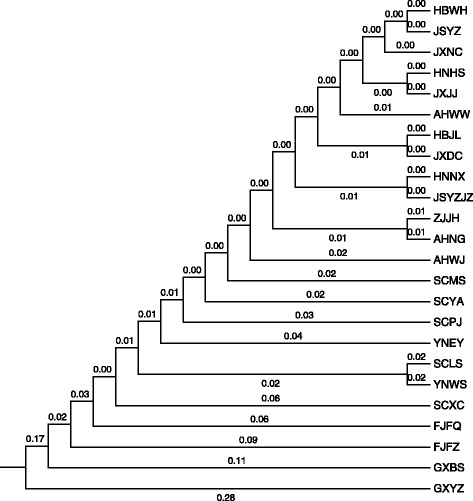
UPGMA cluster analysis of 24 *O. hupensis* populations

UPGMA cluster analysis for the 24 *O. hupensis* populations based genetic distance showed that the populations spread in the landscape of middle and lower reaches of Yangtze River (HBWH, JSYZ, JXNC, HNHS, JXJJ, AHWW, HBJL, JXDC, HNNX, JSYZJZ, ZJJH, AHNG and AHWJ) clustered together first and then gathered with the populations of high mountains (SCMS, SCYA, SCPJ, YNEY, SCLS, YNWS and SCXC), coastal hills (FJFQ and FJFZ) and Karst land form (GXBS and GXYZ) successively (Fig. 3).

## Discussion

*Oncomelania hupensis* is the sole intermediate host for transmitting *Schistosoma japonicum* in mainland China [32], and it is widely distributed in the southern region of the Yangtze River valley. Significant genetic variations have developed in *O. hupensis* from different geographic populations due to their distribution range, complexity of breeding environment and geographical location.

In this research, The genetic differentiation of four different landscape groups of *O. hupensis* were studied through eight screened polymorphic microsatellite DNA loci. This information is pertinent because it further improve our understanding on the effect of genetic diversities on the distribution of *O. hupensis*. This will ultimately help boost our surveillance activities and also strengthen the control of schistosomiasis transmission in China. genetic indices were tested aross eight microsatellite DNA loci. The mean *Fis* value for the 24 populations examined was 0.272, indicating a deficiency of heterozygotes and frequent inbreeding within populations, which is likely due to the small range of activity of *O. hupensis*. A total of 47 microsatellite DNA loci deviated from the Hardy Weinberg Equilibrium demonstrating a serious lack of heterozygotes. Possible explanations that may account for this include: activities of migration and inbreeding, drug pressure, gene mutation and null alleles. However, it is currently unclear which one is the dominant factor contributing to this phenomenon [33]. No significant linkage disequilibrium was found between all pairs of the eight loci, clearly showing the independent behaviour of all loci. Null alleles were found at all eight polymorphic loci. This may be due to: 1) mismatching of primer pairs: mutations in microsatellite DNA sites critical for binding with primers leads to abnormal amplification 2) losses of large alleles: the superiority of short alleles restrict amplification of long fragments or 3) differences in DNA quality: unevenness of templates character obstruct amplification in some loci [26, 31, 34]. Null alleles could implicate genetic diversity parameters for populations such as excess of homozygote individuals, reduction of *Ho* and *He* and increase of genetic distance and *Fis*; moreover, it leads to inaccuracy of parent analysis [30–37].

The abundance of the number of heterozygotes and the amount of genetic information in a population is directly proportional to the *PIC* value [38, 39]. Result shows that *PIC* was greater than 0.5 at every locus, and the mean value (0.947) from all populations was higher than (0.764) obtained from previous result [23]. This signifies that all the eight loci screened were highly polymorphic.

Furthermore, this study reveals that the average *Fst* for all loci was 0.376, which means that 37.6 % of genetic variation was among populations and 72.4 % was among individuals within populations. The analysis of AMOVA displayed that genetic variation among individuals (60.58 %) were far higher than that within populations (26.60 %), while among populations and among groups are (8.04 %) and (4.78 %) respectively. This implies that, genetic diversity is strongly derived from among-individuals rather than among-populations. However, the average *Fst* (0.376) and genetic variation among populations (8.04 %) were higher than values obtained from the previous results (0.048 and 4.8 %) respectively, revealing genetic variation among populations increased along with geographical distance [23]. The Mantel test demonstrated an apparent positive correlation between genetic distance and geographical distance. The genetic structure between geographical populations is embodied with some degree of independence. For example, the geographical distance between the HBWH and JSYZ populations located in the lake region was far, but with low degree of variation. This could possibly be related to the genetic differentiation principally being among individuals within populations rather than among geographic locations for the populations in Lakes and Marshes landscape.

The phylogenetic tree constructed by UPGMA also showed that populations in neighboring geographical locations generally cluster together, which was consistent with the Mantel test results. The cluster sequence of geographical populations showed us that the population from the karst landscape of Guangxi autonomous region maybe the most original one, then the population from the littoral hill part of the Fujian province, the population from the mountainous region of the Sichuan and Yunnan provinces and the population from the region of swamps and lakes in the middle and lower reaches of the Yangtze River, respectively. Regarding as the largest population spread throughout the middle and lower reaches of the Yangtze River [7], the populations from different provinces also crossed cluster, these include, between Hubei and Jiangsu, Hunan and Jiangxi, and Zhejiang and Anhui, which may be as a result of *O. hupensis* spreading along the river within the large population, or gene drifting for surged water flow in the lakes and marshes landscape [34]. Then this branch clustered with the populations of Sichuan and Yunnan province successively. Furthermore, the major branch clustered with the populations of Fujian and Guangxi province in turn, this agrees with the conclusion of four landscape populations relationships from previous studies using SSR-PCR [40] and DNA sequence markers [7, 41, 42].

## Conclusion

This study has shown that the genetic diversity of *O. hupensis*, an important snail intermediate host of *S. japonicum* in China mainly originates from among-individuals rather than among-populations. It also reveals that the populations within subspecies have closer consanguinity than between subspecies in the mass, nevertheless, genetic variations exist within subspecies. These findings further provide important information on genetic structure of *O. hupensis* and strengthen our knowledge about diffusion trend and tracking to the source of *Oncomelania* in mainland China. Ultimately, these findings will help us develop more effective guidelines for controlling the spread and distribution of *Oncomelania* and consequently prevent the transmission of *Schistosomiasis* in China. Our data offers a better understanding of the genetic differentiation of *Oncomelania hupensis,* enhancing our ability to effective and efficient surveillance of Schistosomiasis.
